# Functional properties of resting state networks in healthy full-term newborns

**DOI:** 10.1038/srep17755

**Published:** 2015-12-07

**Authors:** Josepheen De Asis-Cruz, Marine Bouyssi-Kobar, Iordanis Evangelou, Gilbert Vezina, Catherine Limperopoulos

**Affiliations:** 1 Developing Brain Research Laboratory, Children’s National Health System, Washington, D.C., USA, 20010; 2 Division of Diagnostic Imaging and Radiology, Children’s National Health System, Washington, D.C., USA, 20010; 3 Fetal and Transitional Medicine, Children’s National Health System, Washington, D.C., USA, 20010

## Abstract

Objective, early, and non-invasive assessment of brain function in high-risk newborns is critical to initiate timely interventions and to minimize long-term neurodevelopmental disabilities. A prerequisite to identifying deviations from normal, however, is the availability of baseline measures of brain function derived from healthy, full-term newborns. Recent advances in functional MRI combined with graph theoretic techniques may provide important, currently unavailable, quantitative markers of normal neurodevelopment. In the current study, we describe important properties of resting state networks in 60 healthy, full-term, unsedated newborns. The neonate brain exhibited an efficient and economical small world topology: densely connected nearby regions, sparse, but well integrated, distant connections, a small world index greater than 1, and global/local efficiency greater than network cost. These networks showed a heavy-tailed degree distribution, suggesting the presence of regions that are more richly connected to others (‘hubs’). These hubs, identified using degree and betweenness centrality measures, show a more mature hub organization than previously reported. Targeted attacks on hubs show that neonate networks are more resilient than simulated scale-free networks. Networks fragmented faster and global efficiency decreased faster when betweenness, as opposed to degree, hubs were attacked suggesting a more influential role of betweenness hub in the neonate network.

Normal brain function relies on the precise execution of complex neural processes that begins in the 3^rd^ week of gestation and continues into adulthood^1^. Although the brain is continuously remodeling throughout the lifespan, it is most plastic and exceedingly vulnerable during the pre- and perinatal periods^2^. During this time, the brain is rapidly changing and undetected insults may likely lead to long-term neurodevelopmental dysfunction. The ability to objectively and non-invasively assess brain function at these important early stages of neurodevelopment and consequently identify deviations from normal is critical.

Blood oxygen level-dependent (BOLD) signals detected by functional magnetic resonance imaging (fMRI) in the resting brain – or resting state functional connectivity MRI (rs-fcMRI) – may offer critical insights into brain function. Resting state BOLD signals are spontaneous, spatially-coherent, low-frequency (<0.1 Hz) fluctuations detected in the absence of external stimuli^3^. While our understanding of resting-state network physiology remains incomplete, several important observations suggest their assessment may be a potential proxy for direct evaluation of brain function. First, resting state patterns – called resting state networks or RSNs – arise from highly correlated activity within anatomical regions that together form meaningful functional neural systems (i.e. visual processing, memory and attention, among others)^4^. Second, intrinsic brain activity correlates with scores on behavioral tests measured outside the MRI scanner; for instance, signals from the lateral parietal cortex, a component of the executive network, varies with performance in an executive function task^5^. Lastly, functional disconnectivity has been implicated in various neuropsychiatric disorders such as depression^6^, attention deficit hyperactivity disorder^7^, autism spectrum disorder^8^ and schizophrenia^9^. Collectively, these data along with the non-invasive and non-demanding (i.e. minimal patient compliance required) nature of rs-fcMRI makes it a potentially useful clinical tool for evaluating brain function, especially in very young children.

Resting state networks are present and detectable in neonates^10^,^11^,^12^,^13^,^14^,^15^,^16^,^17^ . Primary sensorimotor/auditory/visual networks and putative precursors of higher-order networks such as the default mode network (DMN) and dorsal attention system have been reported. Resting state maps derived using conventional seed-based correlation^3^ and spatial independent component analyses^18^ reveal similar connectivity patterns for term and pre-term neonates scanned at term equivalent age. Differences between these two groups, however, became evident when quantitative approaches were used^19^. Premature infants showed reduced correlation magnitudes, most pronounced in higher order neural systems, and reduced complexity of resting state networks. This recent work suggests that combining rs-fcMRI and quantitative techniques, it is possible to more precisely evaluate resting state activity and quantify deviations from typical brain function.

Graph theory provides a rich, quantitative framework from which to study resting state networks^20^,^21^. The brain is viewed as a complex network and represented as a graph comprised of nodes (or vertices) and edges (or links) – similar to the World Wide Web. Nodes are brain regions of interest (ROIs) and edges, in the case of rs-fcMRI, are the functional connections between nodes. Although a number of recent studies have described resting state activity in newborns, only three studies^16^,^22^,^23^ have characterized newborn RSNs using graph theory. These studies showed that similar to the adult brain^24^,^25^, newborn functional networks exhibit efficient and resilient small world topology. Small-world implies dense local connection between nearby regions (segregation) coupled with the capacity to communicate with distant areas (integration)^26^. Moreover, they reported that important, highly-connected brain areas or ‘hubs’ are mostly sensory/motor regions as opposed to association cortices in adults^16^,^22^.

These studies provide valuable insights into newborn functional networks. However, two out of the three studies also included infants born preterm^16^,^23^. To the best of our knowledge, only one study has used graph measures to characterize properties of RSNs in full-term (infants strictly ≥37 weeks gestational age, GA), healthy, unsedated newborns. Fransson and colleagues, in their 2011 study, evaluated cortical hubs and small world topology in 19 infants^22^. Normative data based on larger samples would provide valuable baseline measures from which to study impaired functional connectivity in high-risk infants. The availability of such well-characterized data would allow us to identify small but important deviations from typical neurodevelopment early which is essential to initiate timely intervention and to minimize long-term developmental impairments. Currently, data derived from larger samples of full-term, healthy newborns are not available.

In this study, we use non-invasive rs-fcMRI and graph theoretic techniques to characterize the functional topology of resting state networks in the healthy neonate brain. We describe key network properties in 60 healthy, full-term neonates (see Methods, Table S1 and Figure S1), namely: small-world topology, degree distributions, community structure and network resilience^27^. To the best of our knowledge, this is the first study to simultaneously examine these network features in a single normative cohort that represents that largest sample of healthy, term neonates evaluated using these measures to date.

First, we describe small-world topology in neonate RSNs, quantify ‘small-worldness’ using the small-world index or *SWI*^28^ and report on cost efficiency or ‘economy’ of these networks^29^,^30^,^31^. We then describe the community structure (i.e. modular organization) of the neonate brain and identify putative brain hubs – regions that are more influential in the network compared to others. We also show how the neonate brain network responds to targeted insult on hubs – defined using degree and betweenness centrality measures – as opposed to random failure of nodes. Lastly, we explore whether removal of degree and betweenness hubs differentially affect the brain’s efficiency.

## Results

### Economical small world properties of neonate functional brain networks

The topology of functional RSNs was examined in 60 healthy, full-term, unsedated newborns (median GA at birth ± MAD: 39.64 ± 0.74 weeks; 35 males) during the neonatal period (median age ± MAD: 12.5 ± 6 days; Table S1). To determine whether neonate brains exhibit small world topology, we computed clustering coefficient, *C*, and characteristic path length, *L*, for each subject and obtained the average, *C*_*neo*_ and *L*_*neo*,_for the group. Clustering coefficient, a measure of network segregation, reflects how well neighbors of a node are connected to each other; characteristic path length, a measure of integration, refers to the average distance between any two nodes in a graph. Relative to random networks, small world networks have high clustering coefficients, *C*_*neo*_ *>> C*_*random*_, and comparable path lengths, *L*_*neo*_* ≥ L*_*random*_^26^.

**Figure 1:**
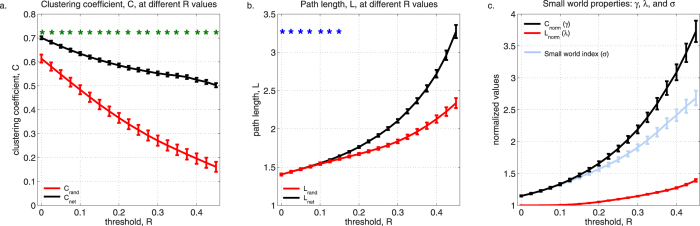
Small-world properties of neonate resting state networks as a function of correlation threshold *R*. (**A**) Group-averaged clustering coefficient for neonates, *C*_*neo*_ (black), and random networks, *C*_*random*_ (red). *C*_*neo*_ is significantly greater than *C*_*random*_ at all thresholds evaluated, *p* < 0.01, Bonferroni corrected (green asterisks). (**B**) Group-averaged characteristic path length for neonates, *L*_*neo*_ (black), and random networks, *L*_*random*_ (red). *L*_*net*_ and *L*_*random*_ are not different (blue asterisks) at threshold range 0 < *R* < 0.175. Degree, *k*, and degree distribution, *p(k)*, of neonate networks are preserved in random networks. (**C**) Small world index, *SWI or σ* (light blue). SWI > 1 (99% CI) for all thresholds tested. Also shown are normalized clustering coefficient (*γ*, black) and normalized characteristic path length (*λ*, red). Here, *γ >>* 1 and *λ ≥ *1. Error bars are standard error of the mean (SEM).

*C*_*neo*_ was significantly greater than *C*_*random*_ at all evaluated correlation thresholds, *R* (*t*-test, *p*_*cor*_ < 0.01, Bonferroni corrected; Fig. 1A), suggesting that neonate brain networks are more ordered compared to random networks. As the correlation threshold *R* increases and graphs become sparser, the value of *C*_*neo*_, as expected, decreases. Figure 1B shows characteristic path length for the neonate brain, *L*_*neo*_, and corresponding random graphs, *L*_*random*_. At *R* values less than 0.175, path lengths were not significantly different between random networks and functional neonate brain networks (*t*-test, *p*_*cor*_ < 0.01, Bonferroni corrected; Fig. 1B). As the threshold increases, more and more nodes have to be traversed to get from one node to the other giving rise to increased path lengths. These findings are consistent with a small-world network.

To quantify small-worldness of normative neonate RSNs, we then measured the small world index (*SWI*, *σ*). This is defined as the ratio between normalized clustering coefficient (*γ* = *C*_*neo*_*/C*_*random*_) and normalized path length (*λ* = *L*_*neo*_*/L*_*random*_)^28^. Small world indices significantly greater than 1 indicate a small world topology. At all thresholds tested, *SWI* was significantly greater than 1 (*SWI* range: 1.15–2.70; 99% confidence interval, *CI*).

To address the influence of variable number of edges at each *R* threshold, the *C*_*neo*_, *L*_*neo*_, and *SWI* were also computed as a function of average graph degree *K* (Fig. S2). Measured values were consistent with a small-world network and the findings above.

**Figure 2:**
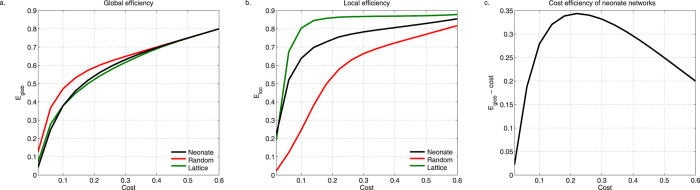
Economy of neonate small world networks. Global (**A**, black; *E*_*glob*_) and local (**B**, black; *E*_*loc*_) efficiency are greater than network cost (*Cost*_*neo*_). Neonate RSNs are cost efficient; *E*_*glob*_ *– cost* is always positive (**C**). Efficiency of neonate networks lie between random (red) and lattice/regular (green) networks.

We then determined economy of small world networks in neonates. We computed global and local efficiency, *E*_*glob*_ and *E*_*loc*_, by averaging efficiency values across all subjects (Fig. 2A and B ). We thresholded graphs at network costs (*Cost*_*neo*_) 0 to 0.6. At these thresholds, *E*_*glob*_ and *E*_*loc*_ exceeded network costs, suggesting cost-efficiency. The maximum value of cost efficiency, *E*_*glob*_ – *Cost*_*neo*_, was at cost = 0.22 (Fig. 2C). Here cost efficiency was 0.3437 (*E*_*glob*_ – *Cost*_*n*eo_: 0.5637–0.2200). At this point, edge density was 22% (881 out of 4005 of all possible edges).

It should also be noted that the efficiency of the neonate brain, both global and local, lay intermediate between lattice (regular) and random networks. This is typical of small world networks.

### Heavy-tailed degree distribution of neonate networks

**Figure 3:**
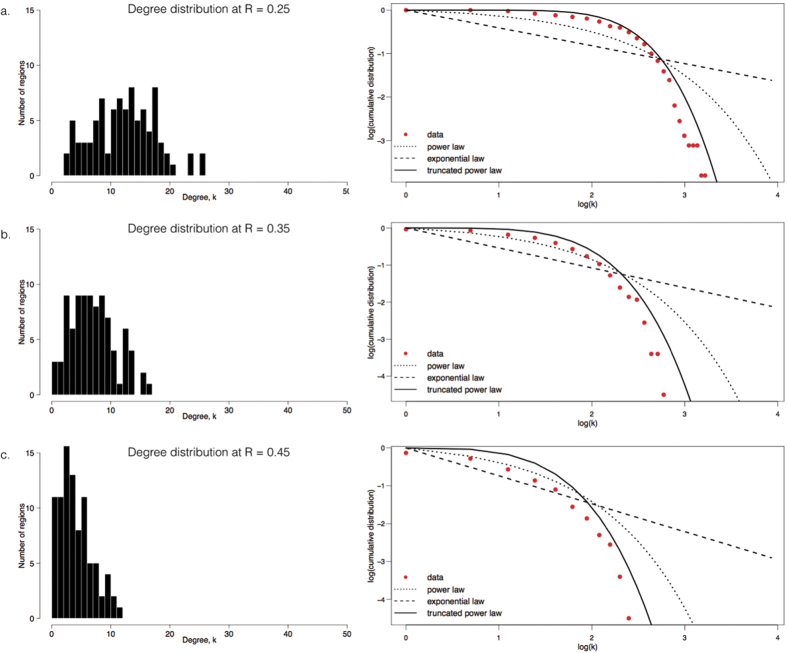
Heavy tailed degree distribution of neonate brains. Right-tailed degree distribution, *p(k)*, of group-averaged neonate networks thresholded at *R* 0.25 (**A**), 0.35 (**B**) and 0.45 (**C**) is shown. This pattern becomes more apparent as the threshold increases and the network gets sparser. (**A**–**C**), left, shows histograms of degree distributions; right panels show log-log plots of cumulative distribution versus degree. Best fit for data (+) is an exponentially truncated power law (··), compared to power law (––) and exponential law (--).

Figure 3 shows the degree distributions, *p(k)*, of group-averaged connectivity matrices thresholded at different *R* values: 0.25, 0.35 and 0.45. The group-averaged degree distribution is heavy-tailed; on the right tail of the distribution, there are a few nodes (hubs) that are more densely connected compared to others. This becomes more evident as the networks become sparser. Individual degree distributions reflect this pattern as well (Fig. S3). The averaged degree distribution follows an exponentially truncated power law, *p(k)* = *k*^*α−1*^*e*^*k/kc*^, as opposed to a power law, *p(k) ~ k*^*−α*^, or an exponential, *p(k)* = *e*^*−(α)(k)*^. The best model was selected using Akaike’s information criterion (AIC; see Table S2 for AIC values).

### Degree and betweenness hubs in the neonate brain

**Figure 4:**
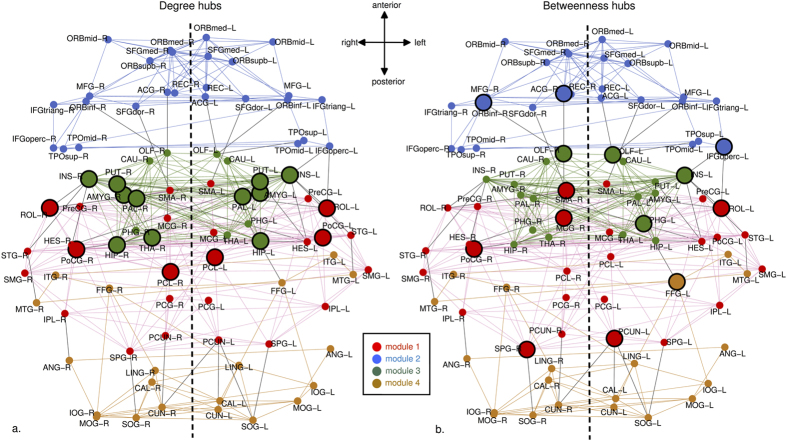
Degree and betweenness hubs. Axial plane shows hubs based on degree (**A**) and betweenness (**B**) as big circles with bold black outlines. Broken lines divide the brain into left and right hemispheres; colors designate modules. Black lines between nodes indicate inter-module connections; intra-module connections have the same color as the nodes they connect. Majority of degree hubs belong to the limbic-paralimbic-subcortical region. Betweenness hubs mostly belong to Module 1 but are relatively more distributed in the brain.

Examining the degree distribution of newborn RSNs show nodes in the right-tail of the degree distribution that are more richly connected compared to other nodes. These are hubs based on degree centrality; we defined degree centrality-based hubs or degree hubs as nodes with degree ≥mean degree ± 1SD. To identify these hubs, we thresholded the averaged functional connectivity matrix at *R* = 0.3159 when the edge density is ~10%, and thus sparse^32^. Degree hubs included (bilateral unless noted): insula, postcentral gyrus, hippocampus, rolandic operculum, amygdala, putamen, pallidum, paracentral lobule, and right thalamus (Fig. 4A; see Table S3 for ROI abbreviations). We also identified hubs based on betweenness centrality or betweenness hubs (betweenness ≥mean betweenness ± 1SD). Betweenness hubs included: bilateral olfactory, left parahippocampal gyrus, left fusiform gyrus, left insula, left rolandic operculum, left inferior frontal gyrus (opercular), left precuneus, right postcentral gyrus, right middle cingulate gyrus, right supplementary motor area, right superior parietal gyrus, right anterior cingulate gyrus, and right inferior orbital gyrus (Fig. 4B). These two centrality measures capture different facets of functional connectivity. Table 1 and 2 lists hubs and their locations. Figure S4 show all 90 nodes arranged by degree and betweenness values.

### Modular organization of the neonate brain

**Figure 5:**
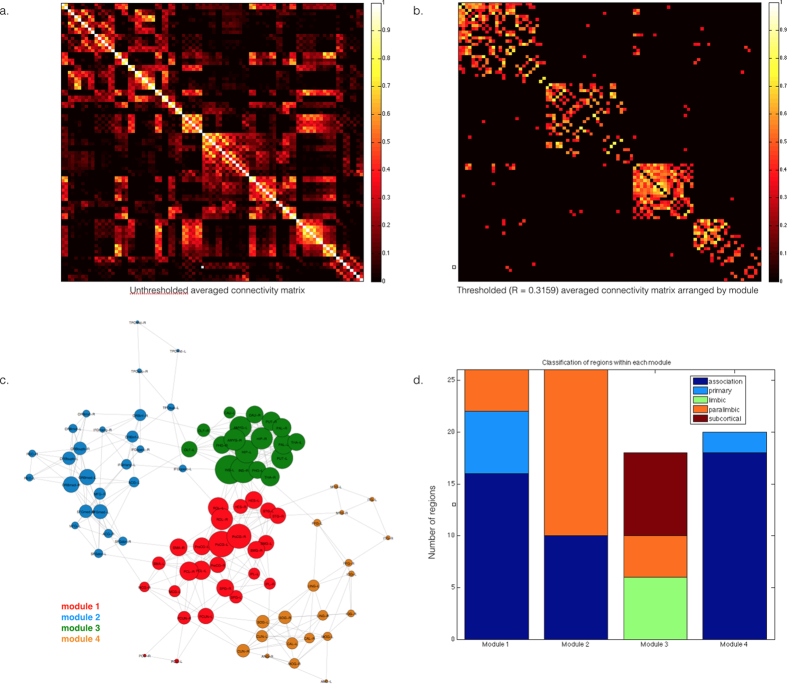
Modular organization of the neonate brain. The Louvain algorithm was used to identify modules. (**A**) shows the unthresholded, averaged connectivity matrix for 60 neonates and (**B**) shows the nodes arranged by module, and by decreasing degree within each module. (**C**) shows the four functional modules identified in the neonate brain. The network is shown using a Kamada-Kawai layout as implemented in the R package igraph; the graph distance between two nodes determines their location in the layout. Node sizes reflect the degree, *k*, of each node. Colors reflect module membership. Classification of node in the modules following Mesulam^60^ is shown in (**D**).

Analysis of community structure of neonate RSNs in our sample revealed a highly modular brain (Figs 4 and 5) with modularity Q = 0.6447. A module refers to a group of densely interconnected nodes; these nodes share a higher number of edges among them than nodes outside their module. Using the Louvain algorithm, we identified four modules in the neonate brain. Module 1 (# of ROIs = 26) comprised primarily of primary, association and paralimbic structures. This included frontoparietal regions such as the somatosensory and primary motor cortices as well as the rest of the somatomotor regions. Middle and posterior cingulate cortices are also Module 1. Module 2 (# of ROIs = 26) comprised paralimbic and association cortices. These included the frontal cortices, temporal poles and anterior cingulate. Module 3 (# of ROIs = 18) comprised mostly limbic, paralimbic, and subcortical structures. The fourth module (# of ROIs = 20) comprised the occipitotemporal cortices; all ROIs in this module are association areas except for the primary visual cortex (calcarine regions). See Table S4 for a list of ROIs per module. The majority of degree hubs belonged to Modules 3 while most betweenness hubs belonged to Module 1.

### Resilience of neonate networks to targeted attacks and random node failure

**Figure 6:**
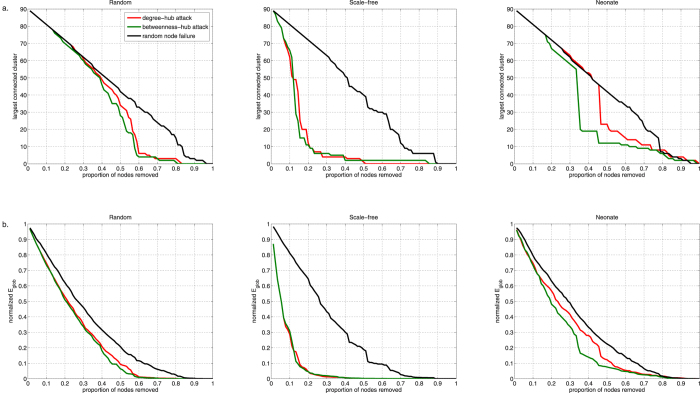
Resilience of resting state networks. Response to targeted removal of degree (red) and betweenness (green) hubs and random removal of nodes (black) are shown for neonate and comparable random and scale-free networks. The effects of node removal to the largest connected cluster (**A**) and global efficiency (**B**) of the network are shown. The neonate brain is more resilient to targeted attack of hubs compared to a scale-free network. Attack on betweenness hubs in the neonate brain decreases global efficiency and the size of the largest cluster faster than attack on degree hubs.

Our data demonstrated that the neonate brain was equally resilient to random failure of nodes as random and scale free networks, but was more resilient to targeted attack of degree or betweenness hubs compared to a scale free network (Fig. 6). In the latter, after 20% of nodes were removed, only 13% of nodes remain connected. A rapid decline in global efficiency was also observed suggesting that information transfer is markedly compromised with removal of hubs in scale-free networks: the remaining network was only 1.75% as efficient as the complete network. In the neonate brain, the majority of nodes remained in the largest connected group – 80% and 74% when degree and betweenness hubs were targeted, respectively – after removal of 20% of nodes. Global efficiency declined, but not to the extent as the scale free network, retaining between 48.1%–56.7% of the original network’s efficiency.

**Figure 7:**
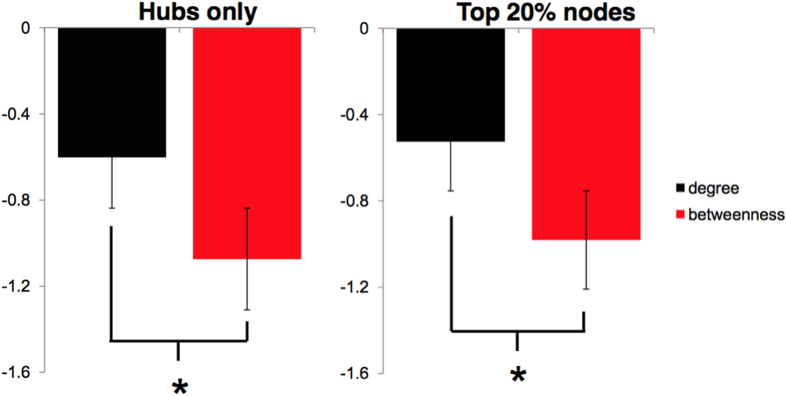
Isolated removal of betweenness and degree hubs. Isolated removal of betweenness hubs cause greater decreases in global efficiency compared to degree hubs (*p* < 0.01). (**A**) shows comparison of hubs (mean ± 1SD) and (**B**) show the top 20% of nodes (n = 18 for both groups).

To further explore the influence of betweenness and degree hubs in the network, we removed individual nodes and recalculated global efficiency of the remaining graph after removal. Isolated removal of hubs (either degree or betweenness) decreased the global efficiency of the network (Fig. 7, Table 3). Elimination of 17 degree and 14 betweenness hubs resulted in an −0.6024 ± 0.3976% (mean ± STD) and −1.0750 ± 0.4380% decrease, respectively, in global efficiency. Isolated removal of betweenness hubs causes a greater drop in efficiency (*t-test, p* < 0.01). We performed a similar analysis for equal-sized group selecting the top 20% (n = 18) of nodes based on degree and betweenness. Since the 18th degree hub is tied among 9 regions *(k* = 12), we used the averaged global efficiency decrease for these nodes. There was significantly greater loss of global efficiency with removal of betweenness hubs (*t-test, p* < 0.01). These findings show that neonate functional brain networks are relatively resilient to targeted node attacks. Moreover, neonate brains withstand removal of degree better than betweenness hubs suggesting a more central role within the network for the latter.

## Discussion

Using fMRI and graph theoretic analyses techniques, we described important features of resting state functional networks in a large sample of healthy, full-term neonates. The following findings were reported, for the first time, in this normative cohort. First, full-term, healthy neonate RSNs met ‘small-worldness’ criteria defined by the small-world index. Related to this, we showed that neonate RSNs are economical: network efficiency is achieved at low cost. Second, these functional networks showed a right-tailed degree distribution, suggesting the presence of highly-connected hubs. Third, we described a slightly more mature pattern of hubs than previously reported. Lastly, we demonstrated that targeted attacks on betweenness hubs, as opposed to degree hubs, resulted in a faster decline in global efficiency and more rapid degeneration of the network.

Neonate brain functional networks in our cohort demonstrated high clustering coefficient (γ >> 1) and average path lengths of ~1 (λ ≥ 1) consistent with a small world topology. Values of γ and λ were comparable to those obtained in neonate structural and functional connectomes reported in previous studies^22^,^23^,^24^,^25^. We also quantified small-world property using the small world index (*σ* = *γ/ λ*); here, *σ* > 1 suggesting an optimal balance between functional brain integration and segregation that is critical for efficient transfer of local and global information. The index we obtained was consistent with previous reports using DTI in neonates^25^,^33^ and in rs-fcMRI studies in adults^25^. These findings are also consistent with SWI measured in younger preterm and full-term infants (35–42 weeks gestational age)^16^. The ability to quantify the small world index in healthy, term neonates provides a quantitative baseline from which to compare network efficiency in high-risk neonatal populations.

Noteworthy, the neonate brain network’s efficiency does not come at the expense of high cost. At all thresholds evaluated, the functional network was found to be cost efficient, where global and local efficiency were greater than network cost. First reported in healthy adults^29^, here we show for the first time that the neonate small world network is economical as well. We reported maximal cost efficiency at edge density of 22%; this is similar to Achard and Bullmore’s report of 21%. They posited that brain networks arose from evolutionary constraints that optimized parallel information processing. If cost-efficiency is an evolutionary adaptation, then it should emerge at the appropriate time of an organism’s life^34^. Throughout gestation and during the first years of life synapses are actively forming and brain volume is growing exponentially^1^. As such, there is likely a compelling need for economy and proper allocation of metabolic resources. Hence, it is not surprising that brain economy is observed in the early postnatal period.

Our neonate functional brain networks showed a right-tailed degree distribution best modeled by an exponentially truncated power law function, unlike a scale-free network, which follows a power law distribution. This is consistent with previous rs-fcMRI studies in adults that used a similar spatial scale of 90 nodes^24^,^35^ and a diffusion temporal imaging or DTI study in neonates using ~500 nodes^36^. Further evidence that neonate RSNs is not scale-free is its better response to targeted removal of nodes. The neonate brain remained more efficient and less fragmented compared to a simulated scale-free network at corresponding removal thresholds. Moreover, as previously pointed out by Achard *et al*.^24^, given that scale-free networks follow a ‘rich get richer’ or ‘preferential attachment’ growth pattern^37^,^38^, we would expect that brain hubs that exist in early infancy would persist until adulthood.

Brain hubs, however, are not static throughout neurodevelopment^39^. Hub configuration changes, gradually transitioning to an adult-like pattern. Previous studies showed that neonatal hubs are mostly primary sensory, motor and limbic regions while heteromodal association areas dominate in adults^16^,^22^,^39^,^40^. Regions we identified as hubs using degree and betweenness centrality measures, and thus central both within and outside their own modules, included the right somatosensory cortex (posterior central gyrus), the left insula, and the left rolandic operculum.

The pattern of our high-degree centrality hubs somewhat varied from what has been previously observed in neonates. We identified unimodal areas such as the somatosensory cortices, similar to previous reports^22^. However, the majority of functional degree hubs were in the subcortical-limbic-paralimbic areas (Table 1 and 2). These regions, while not previously reported as degree hubs in neonates, have been consistently reported in DTI studies in adults. DTI studies in adults characterizing weighted networks identified densely connected structures in the subcortical areas-bilateral putamen, bilateral hippocampus and left thalamus-as high strength nodes (akin to hubs in weighted networks)^33^. A recent structural study in neonates also identified the hippocampus and insula, as well as association areas such as the paracentral lobules, as hubs, which is consistent with our findings in functional networks^36^. These data suggest a structural foundation (i.e. axonal connections) for our rs-fcMRI findings in neonates.

The hubs we identified based on betweenness centrality are in line with previous work in neonates using rs-fcMRI. Similar to the findings of Fransson *et al*.^22^, we also identified SMA, left insula, and right somatosensory cortex. Moreover, Gao *et al*.^16^ also reported the left insula, right inferior frontal (orbital), and left fusiform among the top nodes (top 20 nodes) in neonates. Interestingly, the hubs we identified corresponded better to hubs identified in their 1 year-old cohort. Similarities included bilateral insula, right SMA, bilateral rolandic operculum, left fusiform, bilateral olfactory and bilateral inferior frontal orbital cortices. Gestational age at scan likely contributed to some of the differences in hubs during the neonate period and better concordance with the 1 year old group. We scanned term neonates (GA at birth, range: 37.57–41.86 weeks; GA at scan: 41.50 weeks) whereas previous studies^16^,^22^ included younger neonates (Gao *et al*., GA at birth, range: 35–42 weeks; Fransson *et al*., GA at scan: 39 ±  2 weeks GA). In the case of late pre-term neonates, early exposure to the environment may have led to further changes in hub organization, especially given that this is a period of rapid neural change and reorganization. Additional betweenness hubs we identified have also been consistently reported in adults (using varying centrality measures to identify hubs): left precuneus, right superior parietal gyrus, fusiform gyrus, right middle cingulate cortex, parahippocampal gyrus and right anterior cingulate cortex^24^,^35^,^40^,^41^. Our findings suggest that hub pattern in neonates, while not fully mature – are transitioning to an adult configuration.

Our results also demonstrated that the majority of the high-degree hubs in the neonate brain are localized in the subcortical-limbic-paralimbic region. High-betweenness hubs are mostly in the sensorimotor regions. Our analyses of community structure of neonate networks showed that these regions tend to organize themselves into modules. Modules are nodes that share dense connections with nodes within their group and sparse connections outside their group. We identified four functionally meaningful modules consistent with the findings of van den Heuvel *et al*. 23. This resembles community structure in adults where four to five distinct modules are typically revealed. We observed somatosensory/motor, occipital, fronto-temporal, and limbic-paralimbic-subcortical subsystems. In adults, default mode network such as anterior cingulate, posterior cingulate, precuneus, and temporal cortex are reported to be organized together. We did not observe this in our cohort, suggesting that the while regions implicated in the DMN are beginning to richly connect with other areas (as revealed by their high betweenness values in the hub analyses), the full DMN circuit is still not fully mature.

The response of neonate small world networks to targeted attacks on hubs and random node failure were consistent with previous studies in adults and infants^16^,^24^. Qualitative comparison to adults shows that neonate network disintegrates at a fairly similar rate as adults. Targeted removal of ~45% of degree-hubs and ~35% of betweenness-hubs reduced the largest connected component by 50%; whereas removal of ~40 of nodes caused the network to disintegrate by as much in mature brains^24^. A decrease in global efficiency with targeted node removal was likewise consistent with previous reports^42^. Of note, while the largest connected cluster is noticeably reduced, the effect on global efficiency curves for targeted attacks were slightly less pronounced. Moreover, while network size is decreasing, the global efficiency of the network is somewhat preserved. Conversely, the separation of largest connected cluster plots for betweenness and degree, though, is greater than what is observed in adults. Taken together, these data suggests that hubs identified based on betweenness centrality measures are possibly more influential in the neonate network compared to high degree hubs. The high-degree hubs tended to connect nodes within the same module while the betweenness hubs connected regions that are farther apart. When betweenness hubs are removed, especially in the neonate brain where not all long range connections are fully developed, the brain would be expected to disintegrate faster with removal of the few nodes that traverse modules. In adults, the network can better compensate for removal of betweenness hubs as long range connections are fully developed. This also likely explains why the loss in global efficiency as a result of isolated removal of hubs is greater for the top 20% of betweenness hubs compared to the same number of degree hubs.

This paper attempted to comprehensively describe functional network properties of normal neonate brains relying on BOLD signals. As such, the study shares the same general limitations as other studies based on BOLD functional connectivity. Resting state functional networks reflect temporal coherence between regional signals rather than physical links (i.e. white matter). Studies in adults, however, demonstrated that functional connectivity is constrained by anatomical connectivity^43^. Our study also showed very good consistency with results of previous structural DTI studies, suggesting that rs-fcMRI is a useful technique to map network topology. Nonetheless, ongoing empirical studies that intricately examine the interaction between structural and functional connectivity within this critical period of rapid brain growth and development are needed. In this study, observations were made using a coarse brain parcellation (~90 nodes). Other studies utilized finer spatial scales such as a voxel-based approach. There is no optimal resolution but the efficient small world topology observed in our study is consistent with neonate studies done at different scales.

Here, we described key features of resting state networks in newborns. However, our study limitations deserve mention. First, we were not able to evaluate potential sources of individual differences (i.e. gender, socio-economic status, and mode of delivery) in the measured graph metrics. Gender differences in small-world properties, specifically, have been reported in adults^44^; whether it is evident in newborns is an open question. We hope that our current findings provide a starting point for other studies that examine the relationship between individual factors and network measures. Second, we described representative measures for the properties we described. For instance, we used degree and betweenness centrality to identify hubs. It would also be informative to identify critical brain regions based on other metrics, such as eigenvector centrality^45^, closeness centrality^21^, leverage centrality^45^, and participation coefficient^46^. This is especially important given recent work that suggests that hubs identified using degree-centrality may be confounded by community size^47^. A comparison that includes these other measures, while outside the scope of the current paper, would most certainly be beneficial to our understanding of critical regions in the newborn brains. We hope that our findings motivate future research in neonates that exhaustively examines each of these properties.

In summary, we reported important features of resting state functional networks in a large sample of unsedated healthy, full-term neonates. We demonstrated that neonate resting state functional networks are economical, resilient, and efficient. These networks exhibited small world topology and we quantified this property, for the first time in this neurodevelopmental period in healthy, term neonates, using the small world index. Likewise, we demonstrated the cost-efficiency (‘economy’) of the brain observed in this period. The neonate network organizes itself into functionally meaningful modules. Influential hubs were mostly localized in the sensorimotor and the limbic-paralimbic-subcortical modules. While primary sensory regions were critical hubs in the neonate brain, paralimbic structures (e.g. insula and middle cingulate cortex) and association areas (i.e. precuneus) were already beginning to assume their adult roles as hubs. Moreover, we showed consistency of functional hubs with structural brain hubs. Finally, through random and targeted attacks of these hubs, we showed that the neonate brain is resilient and that betweenness hubs may be more critical than degree hubs at this stage.

## Materials and Methods

### Participants

Seventy two full-term, healthy neonates were recruited from the Children’s National Medical Center as controls in an ongoing prospective study examining brain development in fetuses and infants with congenital heart disease. Sixty neonates (n = 60; median age ± MAD: 12.5 ± 6 days; 35 males) were included in the final analyses; 12 failed to meet motion criteria (described below) and were excluded. All brain MRI studies were reviewed by an experienced pediatric neuroradiologist (GV) and were reported to have structurally normal brains. Table S1 lists subjects’ demographic data. Refer to Table S5 for complete list of exclusion criteria for this study. Parental informed consent was obtained from all participants prior to the study. This study was approved by the Institutional Review Board (IRB) of the Children’s National Health System and the IRB-approved protocol was strictly followed. All experiments were performed in accordance with the regulations and guidelines of the Children’s National Health System IRB.

### Data acquisition

Images were collected on a 3T scanner (Discovery MR750, GE Healthcare, Milwaukee, WI) utilizing an 8 channel infant head coil. T2-weighted fast spin echo MRI was acquired using the following parameters: TR, 2500 ms; TE, 64.49 ms; and voxel size, 0.625 × 1 x 0.625 mm. The acquisition parameters for the gradient-echo planar images (functional images) were: TR, 2000 ms; TE, 35 ms; voxel size, 3.125 × 3.125 × 3 mm ; flip angle, 60˚; field of view, 100 mm; and matrix size: 64 × 64. A total of 200 volumes were collected for approximately seven minutes. To achieve whole brain coverage, around 34 slices (range: 31–36) were obtained per subject.

Infants were scanned while sleeping; none of the neonates were sedated. The infants were fed, swaddled in a warm blanket and immobilized using an infant vacuum pillow. Ear protection for resulting MRI noise was provided using silicone ear plugs and adhesive ear muffs. Physiologic state of subjects (heart rate and oxygen saturation) was monitored by a nurse for the duration of the study.

### Preprocessing of functional images

Images were preprocessed using the AFNI software package^48^ unless otherwise noted. After exclusion of the first four echo-planar images to allow for signal stabilization and removal of large spikes in the data^49^, slice-dependent temporal offsets and image inhomogeneities (using ANTS N4 tool^50^) were corrected. Functional volumes were rigidly registered to a base EPI volume, normalized to a global mode of 1000^51^, aligned to the T2-weighted structural image, normalized – along with the structural image – to a neonate anatomical template^52^, and smoothed (full-width-at-half-maximum = 4 mm).

To minimize the influence of extraneous signals on the spontaneous BOLD fluctuations, nuisance signals from white matter and ventricles, along with motion parameters and their derivatives were regressed out of the voxel-wise time series.

To reduce the possible effects of spurious head motion on the BOLD signal, volumes with high motion – those with frame-wise displacement >0.3 mm – were excluded (‘censored’ or ‘scrubbed’)^49^,^53^. Volumes with a high fraction of BOLD signal outliers (greater than 10% voxels; implemented using AFNI’s 3dToutcount) were also removed. Subjects who failed to meet these criteria were removed from the analyses. The time series were then band-pass filtered selecting signals in the range 0.01 < *f* < 0.1. Censoring, nuisance regression and band-pass filtering were simultaneously performed. Residual time series for all voxels within the 90 cortical and subcortical regions of interest (ROIs) were averaged to represent the BOLD signal for that particular region. These ROIs are based on the Automated Anatomical Labeling (AAL)^54^ parcellation and mapped to neonates and infants by Shi *et al*.^52^.

The average number of volumes per subjects was 173 ± 15 frames (range: 148–196) corresponding to ~5.8 minutes of data. There were, on average, 23 ± 15 volumes (~11.73% of collected data) of data removed.

### Graph analysis

See Figure S5 for summary of graph analysis.

#### Graph formation

Each subject’s graph, *G*_*neo*_, was composed of 90 vertices or nodes derived from the 90 ROIs. The correlation between all pairs of nodes (edges or links) were computed giving rise to a 90 × 90 resting state functional connectivity matrix, *M*_*neo*_, for each subject. *M*_*neo*_ was thresholded and then binarized such that all cells that met the threshold were set to 1 and the rest to 0. For each subject, this yielded an undirected binary graph, *G*_*neo*_, per threshold. For analyzing small-world properties, we used correlation (*R*) and degree (*K*) threshold values. Correlation, *R*, values ranged from 0 to 0.45 (intervals of 0.025). The choice of threshold affects computed graph metrics^55^. It is thus useful to evaluate brain networks at different correlation values. As the threshold *R* is increased, connections between nodes are reduced or the graphs become increasingly sparse. The graph eventually fragments: nodes disconnect from the rest of the graph. We empirically set the upper limit of R values to 0.45 such that there will be one largest connected component for each individual graph, rather than multiple clusters. At 0.45, individual subjects’ *M*_*neo*_ remained at least 90% connected^25^. The upper limit of evaluated thresholds was also constrained by the average degree (*K*_*ave*_, average number of connections nodes have) of the group averaged connectivity matrix. The group-averaged matrix was obtained by averaging individual correlation matrices (60 × *M*_*neo*_), thresholding the resulting 90 × 90 average graph, and binarizing the network to yield *G*_*ave*_. At *K*_*ave*_ < log(number of nodes) ~4.5, estimated small world properties become unreliable^24^. At *R* = 0.45, *K*_*ave*_ for the group-averaged graph is 4.5. At a particular threshold, the number of edges per graph for each subject will not be consistent. To determine the influence of variable number of edges on our results, we also evaluated subjects’ graphs at different degree (*K*) thresholds. We evaluated networks at the range 6 < *K* < 42. Beginning *K* = 12, the largest connected cluster is always at least 90% for each subject.

Each subject will have 19 *R* and 13 *K* thresholded graphs. Small-world properties are typically evaluated relative to a random network. Here, we generated 100 random networks for each subject network, each one preserving the degree distribution of the original network. Thus, each subject will have 101 networks per threshold.

To determine economy of neonate networks, we evaluated the relationship between network efficiency and cost. Thus, in this analyses, we thresholded individual networks based on their cost (*Cost*_*neo*_). Network cost is the ratio of actual edges in the network to the number of possible edges. We evaluated networks at costs in the range of 0.02 to 0.60 (intervals of 0.04)^29^. We compared neonate network efficiency to efficiency of random and regular (or lattice) networks. For this analysis, we generated 20 random and 20 lattice networks for each subject at each cost threshold such that each subject will have 41 networks per threshold.

#### Small-world efficiency and economy analyses

Graph metrics were computed using the publicly available Brain Connectivity Toolbox found here: https://sites.google.com/site/bctnet/21. The following topological properties of neonate resting state functional brain networks were assessed: clustering coefficient (*C*), path length (*L*), small world index (*SWI*), global efficiency (*E*_*glob*_) and local efficiency (*E*_*loc*_). These measures were computed for each subject and then averaged.

1. Clustering coefficient, *C*, describes the tendency of neighbors of a node to cluster together. Small world networks have high clustering coefficient relative to a random graph, *C*_*neo*_ >> *C*_*random*_, and normalized clustering coefficient, *γ* >> 1, where *γ* = *C*_*neo*_/*C*_*random*_.
2. Characteristic path length, *L*, is the average shortest distance between any two nodes in a graph. Normalized path length, λ, is the ratio between a subject’s average path length, *L*_*neo*_, and that of a random network, *L*_*random*_. In small world networks, path length *L*_*neo*_ ≥ *L*_*random*_. Both *C* and *L* were computed from the largest connected component of the graph similar to van den Heuvel (18).
3. Small world index, *SWI* (*σ*), is a scalar value that quantifies the ‘small-world-ness’ of a network^28^; σ > 1, where σ = *γ/*λ, indicates a small world network.
4. Global efficiency, *E*_*gl*ob_, reflects how well information is transferred in a network; it is inversely related to path length (*1/L*)^56^. Global efficiency of small world networks is intermediate that of random and regular networks (*E*_*globR*_* > E*_*glob*_* > E*_*globL*_). Cost efficiency is the difference between global efficiency and network cost, *E*_*glob*_–*Cost*_*neo*_; this value is positive in an economical network^29^,^57^.
5. Local efficiency, *E*_*loc*_, is related to clustering coefficient^56^. *E*_*loc*_ measures how well neighbors of a node communicate with each other after the node is removed. Small world network local efficiency lies between random and lattice networks, *E*_*locR*_* > E*_*loc*_* > E*_*locL*_.

#### Hub and modularity analyses

We evaluated the degree distribution, *p(k)*, of individual and group-averaged functional networks. *p(k)* refers to the probability of a node *i* to have degree *k*. Real-world complex networks typically show a heavy-tailed degree distribution with hubs, or influential nodes, occupying the right side of the distribution. The best fit for the distribution was selected using Akaike’s information criterion implemented using the R package brainwaver here: http://cran.r-project.org/web/packages/brainwaver/index.html^24^. We identified hubs in the group-averaged networks, *G*_*ave*_. We used two centrality measures to define hubs: degree and betweenness. We defined degree hubs as nodes with degree ≥ mean degree ± 1SD. Betweenness refers to the number of shortest paths that pass through a node. Nodes with betweenness values ≥ mean betweenness ± 1SD were identified as betweenness hubs. Results were shown for the *G*_*ave*_ thresholded at *R* = 0.3159; at this threshold the group averaged connectomes is sparse (edge density = 10%) and fully connected. To visualize hubs in space, we identified the centers of mass of the 90 ROIs using AFNI’s 3dCM and plotted the x-y coordinates of the nodes (axial view).

To assess the modular organization of *G*_*ave*_, we used the Louvain method^58^ for community detection. The algorithm was iterated 10,000 times and fine-tuned thereafter. Results are shown at the same threshold as above but module partitions were fairly consistent across thresholds 0.15 ≤ *R* ≤ 0.35.

#### Resilience analyses

To determine resilience of neonate brain networks, we compared its response to random and targeted node attacks to comparable random and scale-free networks. For random failure of nodes, a node was randomly removed and the largest connected component^24^,^59^ and global efficiency^16^,^42^ were recalculated. For targeted attacks, nodes were removed one by one starting from the node with the highest degree or betweenness.

We also computed global efficiency after isolated removal of nodes and compared the impact of removal of degree versus betweenness hubs. One node and all its connections were removed and then global efficiency is recalculated for the remaining graph.

## Additional Information

**How to cite this article**: De Asis-Cruz, J. *et al*. Functional properties of resting state networks in healthy full-term newborns. *Sci. Rep*. **5**, 17755; doi: 10.1038/srep17755 (2015).

## Supplementary Material

Supplementary Information

## Figures and Tables

**Table 1:** Degree hubs and their location.

Degree hub	x	y	z
INS-L	22	1	5
INS-R	−23	2	3
PoCG-L	30	16	32
PoCG-R	−26	18	34
HIP-L	16	16	−5
ROL-R	−33	9	11
HIP-R	−17	17	−5
AMYG-L	16	5	−9
AMYG-R	−16	6	−10
PUT-L	16	3	4
ROL-L	31	9	12
PAL-L	12	6	4
PCL-L	5	20	43
PCL-R	−5	24	42
PUT-R	−17	3	4
PAL-R	−13	7	2
THA-R	−9	16	7

Coordinates are in the space of the neonate atlas.

**Table 2:** Betweenness hubs and their location.

Betweenness hub	x	y	z
OLF-L	6	−4	−6
PHG-L	13	12	−13
FFG-L	21	26	−14
INS-L	22	1	5
ROL-L	31	9	12
IFGoperc-L	31	−6	13
PCUN-L	6	40	31
PoCG-R	−26	18	34
MCG-R	−6	11	25
SMA-R	−5	5	39
SPG-R	−14	42	41
ACG-R	−6	−18	10
ORBinf-R	−24	−16	−7
OLF-R	−6	−4	−6

Coordinates are in the space of the neonate atlas.

**Table 3:** Change in global efficiency after isolated removal of nodes.

	Brain regions	Δ Eglob (%)		Brain regions	Δ Eglob (%)		Brain regions	Δ Eglob (%)
1	PHG-L	−2.00	31	SPG-L	−0.37	61	ORBsupb-R	0.03
2	OLF-L	−1.73	32	AMYG-L	−0.35	62	SFGdor-L	0.06
3	INS-L	−1.53	33	HES-R	−0.33	63	CUN-L	0.10
4	FFG-L	−1.39	34	SMA-L	−0.32	64	ORBsupb-L	0.17
5	ROL-L	−1.17	35	PreCG-L	−0.31	65	IFGoperc-R	0.17
6	PoCG-R	−1.11	36	SOG-R	−0.30	66	FFG-R	0.17
7	SPG-R	−0.97	37	HIP-R	−0.30	67	CAL-L	0.21
8	INS-R	−0.93	38	PUT-L	−0.30	68	MTG-R	0.22
9	SMA-R	−0.84	39	PreCG-R	−0.29	69	MOG-R	0.22
10	IFGoperc-L	−0.84	40	SMG-R	−0.28	70	MOG-L	0.23
11	MCG-R	−0.81	41	PHG-R	−0.26	71	LING-R	0.25
12	PoCG-L	−0.80	42	SMG-L	−0.24	72	MTG-L	0.25
13	ROL-R	−0.78	43	PAL-L	−0.23	73	MFG-L	0.28
14	OLF-R	−0.75	44	LING-L	−0.22	74	IOG-L	0.28
15	ORBinf-R	−0.75	45	SOG-L	−0.19	75	CAL-R	0.29
16	STG-R	−0.74	46	SFGdor-R	−0.17	76	ANG-R	0.30
17	STG-L	−0.73	47	SFGmed-L	−0.15	77	IOG-R	0.33
18	THA-R	−0.63	48	THA-L	−0.14	78	ORBmid-L	0.35
19	PCUN-L	−0.59	49	PUT-R	−0.14	79	ORBmid-R	0.36
20	ACG-R	−0.57	50	PAL-R	−0.14	80	IFGtriang-R	0.39
21	PCL-L	−0.55	51	CAU-R	−0.12	81	TPOsup-R	0.39
22	TPOsup-L	−0.51	52	MCG-L	−0.11	82	ITG-L	0.43
23	IPL-R	−0.49	53	SFGmed-R	−0.07	83	ITG-R	0.50
24	HES-L	−0.48	54	IFGtriang-L	−0.06	84	PCG-L	0.50
25	PCL-R	−0.46	55	IPL-L	−0.03	85	REC-L	0.53
26	HIP-L	−0.43	56	CAU-L	−0.01	86	REC-R	0.53
27	ACG-L	−0.43	57	MFG-R	0.00	87	PCG-R	0.59
28	ORBinf-L	−0.42	58	ORBmed-R	0.01	88	TPOmid-L	0.68
29	PCUN-R	−0.40	59	CUN-R	0.01	89	ANG-L	0.72
30	AMYG-R	−0.39	60	ORBmed-L	0.03	90	TPOmid-R	0.88

Nodes are arranged from nodes that caused the greatest reduction in global efficiency.
