# Supplementary material for: Functional properties of resting state networks in healthy full-term newborns
Source: Sci Rep. 2015 Dec 7;5:17755. doi: 10.1038/srep17755 (PMC4671028; doi:10.1038/srep17755)
Supplement: Supplementary Information [file srep17755-s1.pdf]

Supplementary Information

Functional properties of resting state networks in healthy full-term newborns

Authors: Josepheen De Asis-Cruz, M.D., Ph.D[1], Marine Bouyssi-Kobar, M.S.[1], Iordanis Evangelou, D.Phil[1], Gilbert Vezina, M.D.[2], and Catherine Limperopoulos Ph.D.[1,2,3,*]

[1] Advanced Pediatric Brain Imaging Research Laboratory, Children's National Health System, Washington, D.C., USA, 20010
[2] Division of Diagnostic Imaging and Radiology, Children's National Health System, Washington, D.C., USA, 20010
[3] Fetal and Transitional Medicine, Children's National Health System, Washington, D.C., USA, 20010

**** Corresponding author:***

Catherine Limperopoulos, Ph.D.
Advanced Pediatric Brain Imaging Research Laboratory
Children's National Health System
111 Michigan Avenue, NW Washington, D.C 20010
202 476 5293
CLimpero@childrensnational.org

Supplementary figure legends

Figure S1. Age distribution of n = 60 neonates. Gestational ages of neonates (GA) at scan (A) and at birth (B) are shown, along with weeks of life at scan (C). (D) shows GA at scan plotted against GA at birth. Green points in (A) and (B) show distribution of ages within each histogram bin.

Figure S2. Small-world properties of neonate resting state networks as a function of degree, *K*. Findings are consistent with correlation thresholded networks shown in Figure 1. (A) shows the C_{neo} (black) and C_{random} (red). C_{neo} is significantly greater than C_{random} at degree range $42 > K > 6$, $p < 0.01$, Bonferroni corrected (green asterisks). (B) shows L_{neo} (black) and L_{random} (red). L_{net} and L_{random} are not different at $K = 42$ (green triangles). All generated random networks preserve the degree, *k*, and degree distribution, *p(k)* of the original networks. (C) shows small world index, SWI or σ (light blue). SWI > 1 (99% CI) for all thresholds tested. Also shown are γ (black) and λ (red). Here, γ >> 1 and λ ≥ 1. Error bars refer to SEM.

Figure S3. Right-tailed individual degree distributions. (A)-(C), left, shows the mean degree distribution for n = 60 neonates (blue) superimposed on individual degree distributions (gray). The histograms on the right panels show the same data as the blue plots on the left.

Figure S4. Hubs based on degree and betweenness centrality. Node-specific degree (A) and betweenness (B) values are shown for all 90 ROIs. Red lines mark mean, dashed red lines mark mean ± 1SD.

Figure S5. Summary of graph analysis.

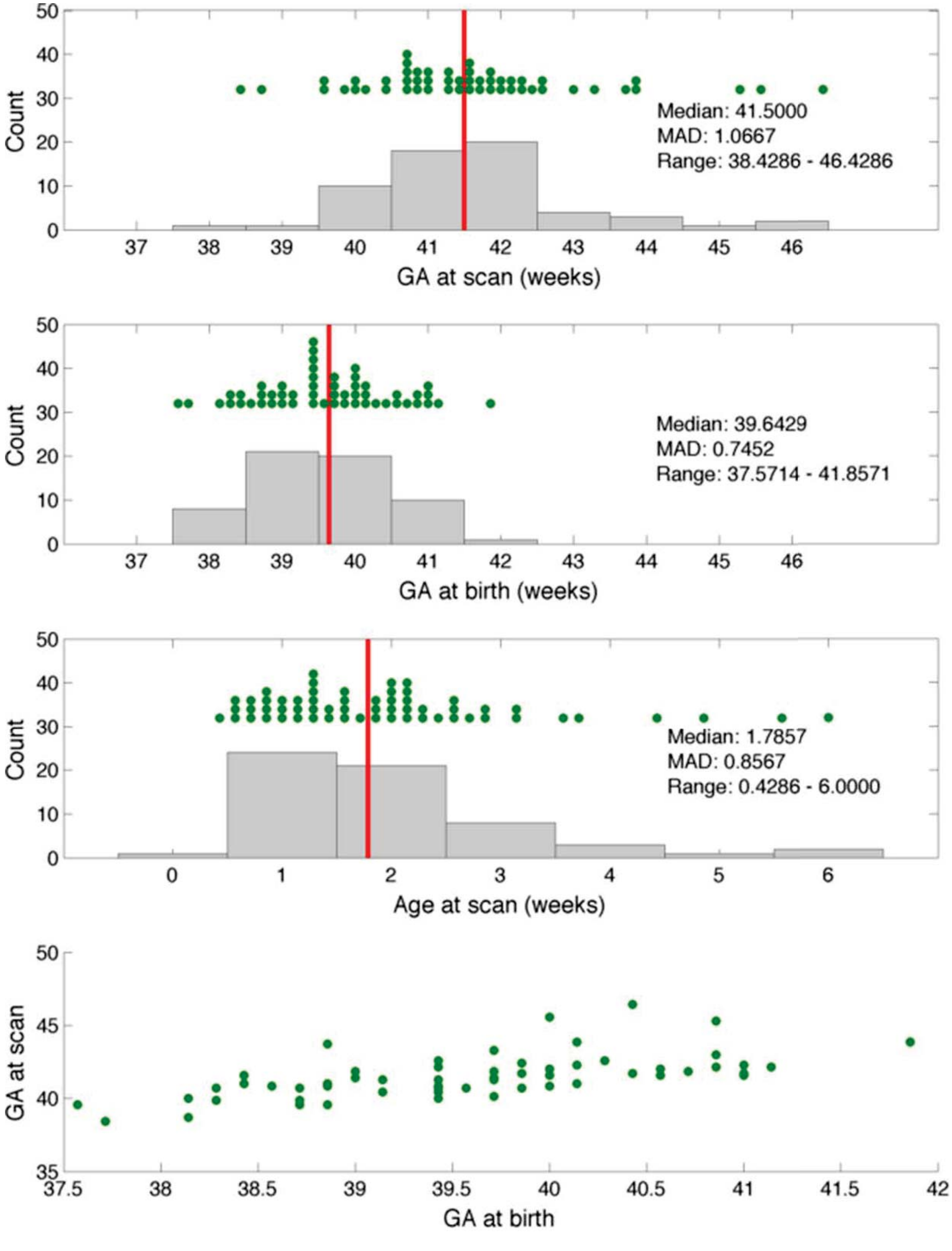
Count
Median: 41.5000
MAD: 1.0667
Range: 38.4286 - 46.4286
GA at scan (weeks)
Count
Median: 39.6429
MAD: 0.7452
Range: 37.5714 - 41.8571
GA at birth (weeks)
Count
Median: 1.7857
MAD: 0.8567
Range: 0.4286 - 6.0000
Age at scan (weeks)
GA at scan
GA at birth

Figure S1

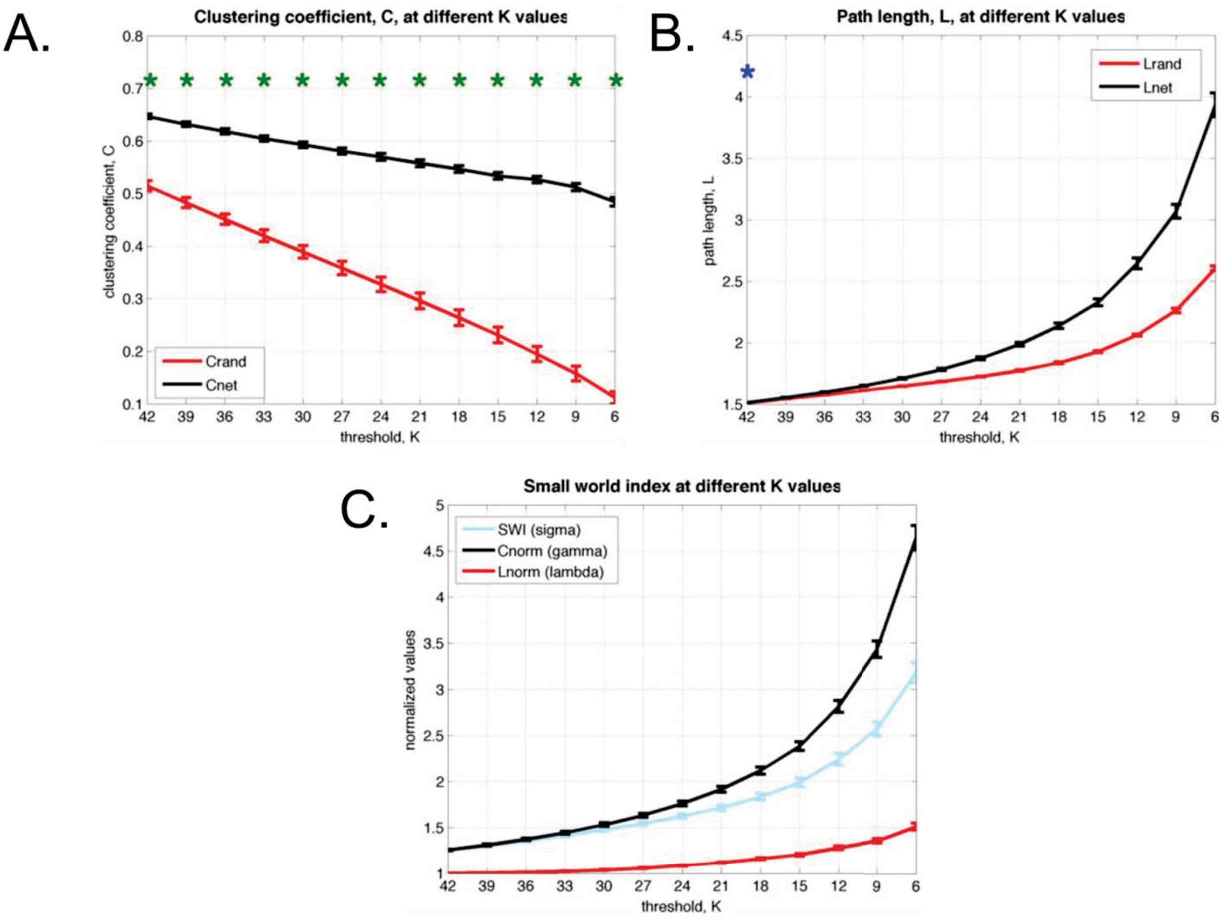
A.
Clustering coefficient, C, at different K values
clustering coefficient, C
threshold, K
Crand
Cnet
B.
Path length, L, at different K values
path length, L
threshold, K
Lrand
Lnet
C.
Small world index at different K values
normalized values
threshold, K
SWI (sigma)
Cnorm (gamma)
Lnorm (lambda)

Figure S2

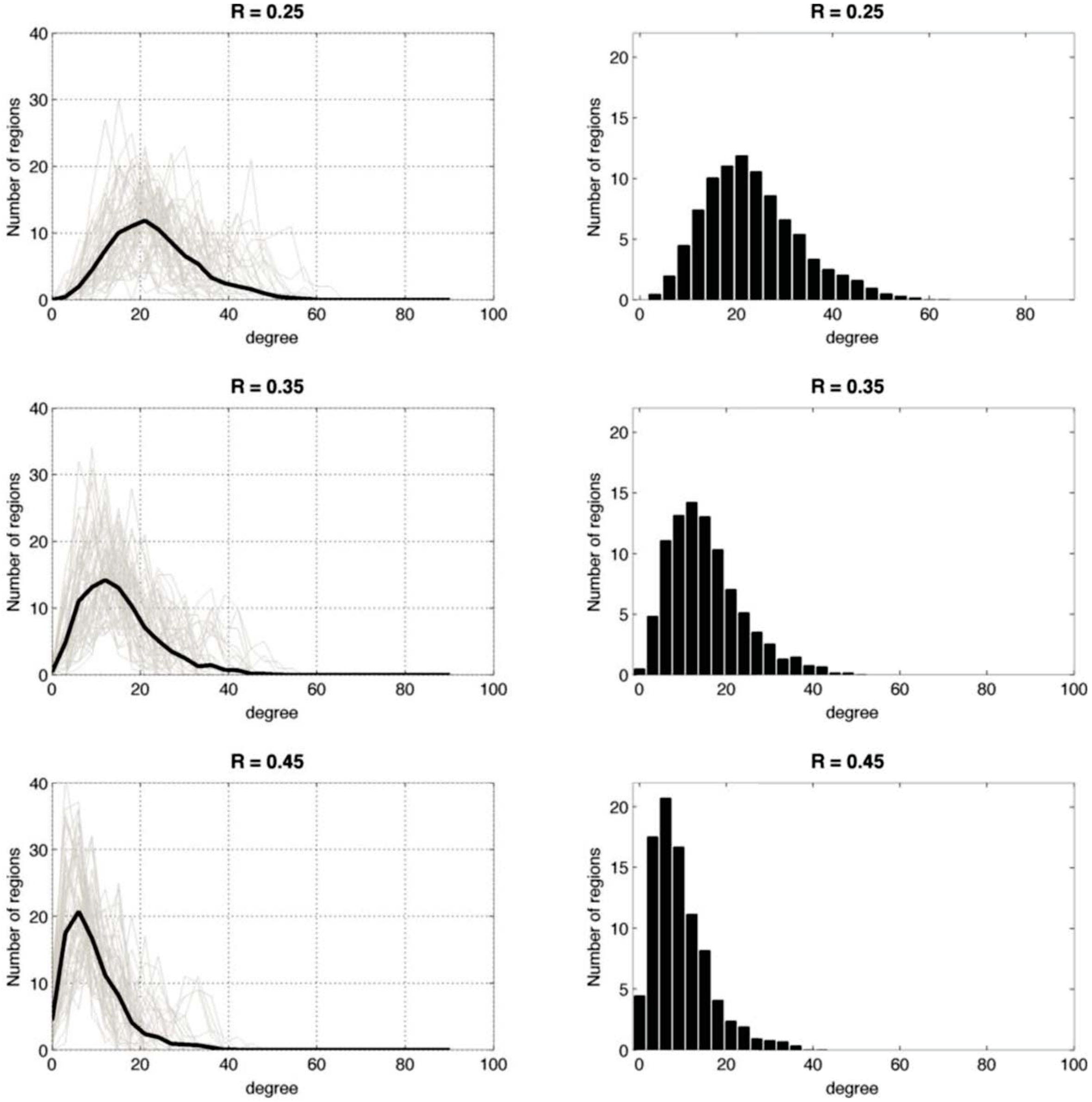

Figure S3

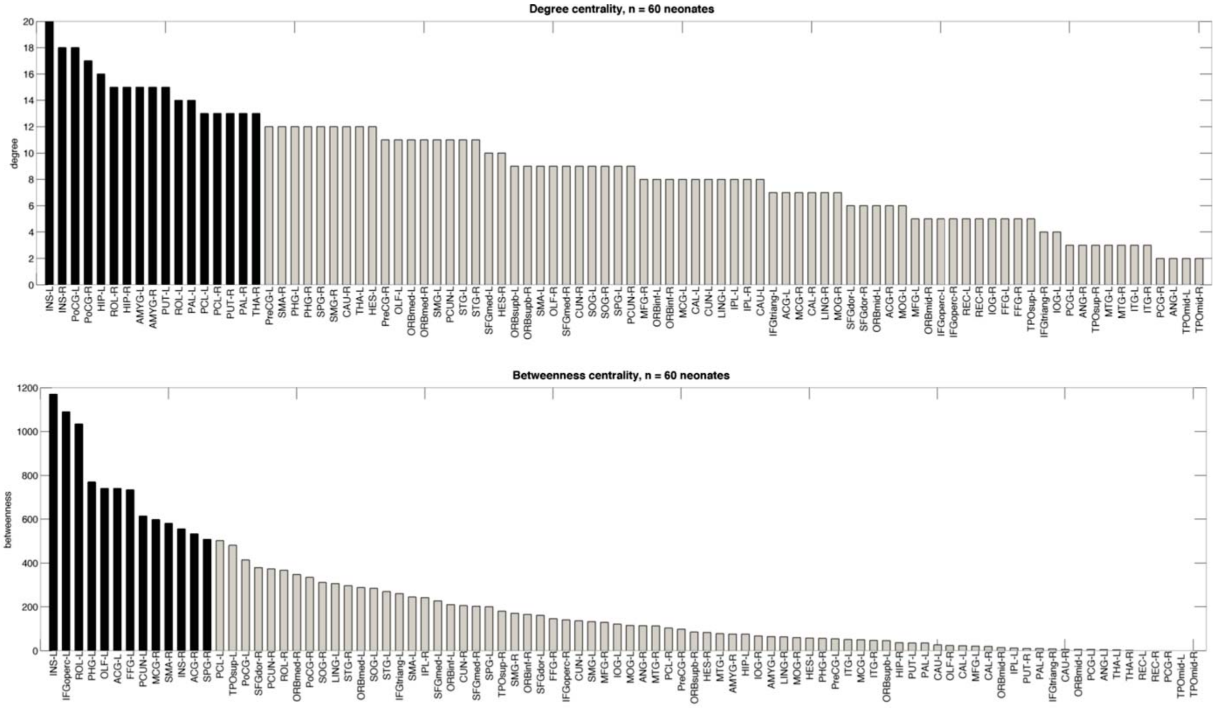
Degree centrality, n = 60 neonates
degree
0
2
4
6
8
10
12
14
16
18
20
INS-L
INS-R
PoCG-L
PoCG-R
HIP-L
ROL-R
HIP-R
AMYG-L
AMYG-R
PUT-L
ROL-L
PAL-L
PCL-L
PCL-R
PUT-R
PAL-R
THA-R
PreCG-L
SMA-R
PHG-L
PHG-R
SPG-R
SMG-R
CAU-R
THA-L
HES-L
PreCG-R
OLF-L
ORBmed-L
ORBmed-R
SMG-L
PCUN-L
STG-L
STG-R
SFGmed-L
HES-R
ORBsupb-L
ORBsupb-R
SMA-L
OLF-R
SFGmed-R
CUN-R
SOG-L
SOG-R
SPG-L
PCUN-R
MFG-R
ORBinf-L
ORBinf-R
MCG-L
CAL-L
CUN-L
LING-L
IPL-L
IPL-R
CAU-L
IFGtriang-L
ACG-L
MCG-R
CAL-R
LING-R
MOG-R
SFGdor-L
SFGdor-R
ORBmid-L
ACG-R
MOG-L
MFG-L
ORBmid-R
IFGoperc-L
IFGoperc-R
REC-L
REC-R
IOG-R
FFG-L
FFG-R
TPOsup-L
IFGtriang-R
IOG-L
PCG-L
ANG-R
TPOsup-R
MTG-L
MTG-R
ITG-L
ITG-R
PCG-R
ANG-L
TPOmid-L
TPOmid-R
Betweenness centrality, n = 60 neonates
betweenness
0
200
400
600
800
1000
1200
INS-L
IFGoperc-L
ROL-L
PHG-L
OLF-L
ACG-L
FFG-L
PCUN-L
MCG-R
SMA-R
INS-R
ACG-R
SPG-R
PCL-L
TPOsup-L
PoCG-L
SFGdor-R
PCUN-R
ROL-R
ORBmed-R
PoCG-R
SOG-R
LING-L
STG-R
ORBmed-L
SOG-L
STG-L
IFGtriang-L
SMA-L
IPL-R
SFGmed-L
ORBinf-L
CUN-R
SFGmed-R
SPG-L
TPOsup-R
SMG-R
ORBinf-R
SFGdor-L
FFG-R
IFGoperc-R
CUN-L
SMG-L
MFG-R
IOG-L
MOG-L
ANG-R
MTG-R
PCL-R
PreCG-R
ORBsupb-R
HES-R
MTG-L
AMYG-R
HIP-L
IOG-R
AMYG-L
LING-R
MOG-R
HES-L
PHG-R
PreCG-L
ITG-L
MCG-L
ITG-R
ORBsupb-L
HIP-R
PUT-L
PAL-L
CAU-L
OLF-R
CAL-L
MFG-L
CAL-R
ORBmid-R
IPL-L
PUT-R
PAL-R
IFGtriang-R
CAU-R
ORBmid-L
PCG-L
ANG-L
THA-L
THA-R
REC-L
REC-R
PCG-R
TPOmid-L
TPOmid-R

Figure S4

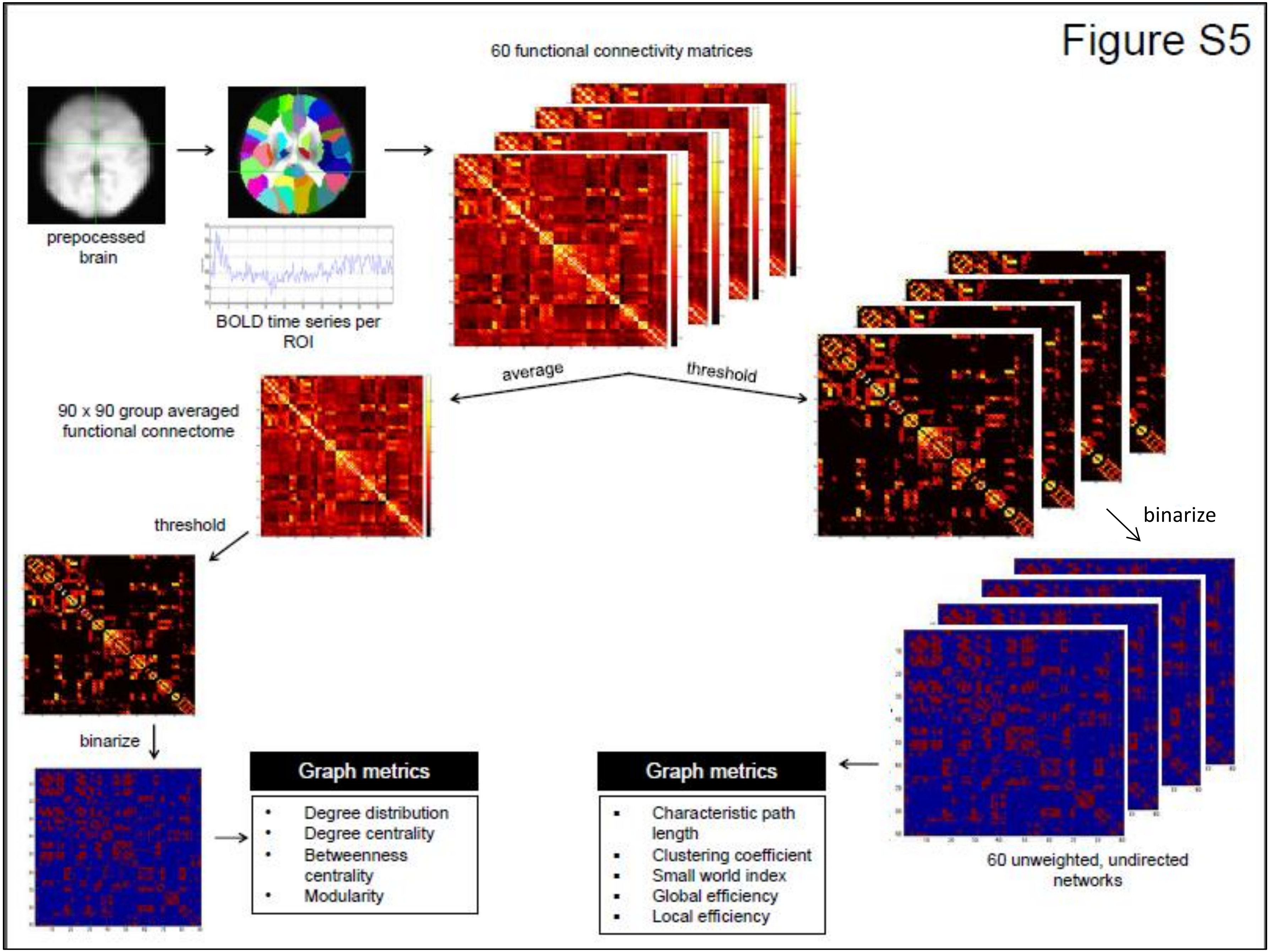
Figure S5
60 functional connectivity matrices
prepocessed brain
BOLD time series per ROI
average
threshold
90 x 90 group averaged functional connectome
threshold
binarize
binarize
60 unweighted, undirected networks
Graph metrics
Degree distribution
Degree centrality
Betweenness centrality
Modularity
Graph metrics
Characteristic path length
Clustering coefficient
Small world index
Global efficiency
Local efficiency

TABLE S1. Demographic data for 60 neonates.

	Median ± MAD	Range
**GA at birth (weeks)**	39.64 ± 0.74	37.57 - 41.86
**GA at scan (weeks)***	41.50 ± 1.07	38.43 - 46.43
**Age at scan (days)**	12.50 ± 6.00	0.43 - 42.00
**Birth weight (kg)****	3391.50 ± 261.47	2594 - 3968
**Apgar 1**	8.00 ± 0.68	1 - 9
**Apgar 5**	9.00 ± 0.25	5 - 9

* All subjects, except for 3, are neonates (< 4 weeks old).
** Birth weight not available for 2 infants.
*** Apgar scores not available for 14 infants.

TABLE S2. AIC values for degree distribution models

Model	AIC* Values		
	R = 0.25	*R* = 0.35	*R* = 0.45
**Power law**	780	628	466
**Exponentially truncated power law**	560	492	403
**Exponential**	639	544	453

* AIC: Akaike Information Criterion

TABLE S3. Regions of interest (ROIs). Abbreviations used by Shi et al. (2011) were adopted.

Region	Abbreviation
Precentral gyrus	PreCG
Superior frontal gyrus (dorsal)	SFGdor
Orbitofrontal cortex (superior)	ORBsupb
Middle frontal gyrus	MFG
Orbitofrontal cortex (middle)	ORBmid
Inferior frontal gyrus (opercular)	IFGoperc
Inferior frontal gyrus (triangular)	IFGtriang
Orbitofrontal cortex (inferior)	ORBinf
Rolandic operculum	ROL
Supplementary motor area	SMA
Olfactory	OLF
Superior frontal gyrus (medial)	SFGmed
Orbitofrontal cortex (medial)	ORBmed
Rectus gyrus	REC
Insula	INS
Anterior cingulate gyrus	ACG
Middle cingulate gyrus	MCG
Posterior cingulate gyrus	PCG
Hippocampus	HIP
Parahippocampal gyrus	PHG
Amygdala	AMYG
Calcarine cortex	CAL

Region	Abbreviation
Cuneus	CUN
Lingual gyrus	LING
Superior occipital gyrus	SOG
Middle occipital gyrus	MOG
Inferior occipital gyrus	IOG
Fusiform gyrus	FFG
Postcentral gyrus	PoCG
Superior parietal gyrus	SPG
Inferior parietal lobule	IPL
Supramarginal gyrus	SMG
Angular gyrus	ANG
Precuneus	PCUN
Paracentral lobule	PCL
Caudate	CAU
Putamen	PUT
Pallidum	PAL
Thalamus	THA
Heschl gyrus	HES
Superior temporal gyrus	STG
Temporal pole (superior)	TPOsup
Middle temporal gyrus	MTG
Temporal pole (middle)	TPOmid
Inferior temporal gyrus	ITG

TABLE S4. Regions of interest within each module and their tissue classification*.

Module 1	Class	Module 2	Class	Module 3	Class	Module 4	Class
PreCG-L	primary	SFGdor-L	association	OLF-L	limbic	CAL-L	primary
PreCG-R	primary	SFGdor-R	association	OLF-R	limbic	CAL-R	primary
ROL-L	association	ORBsupb-L	paralimbic	INS-L	paralimbic	CUN-L	association
ROL-R	association	ORBsupb-R	paralimbic	INS-R	paralimbic	CUN-R	association
SMA-L	association	MFG-L	association	HIP-L	limbic	LING-L	association
SMA-R	association	MFG-R	association	HIP-R	limbic	LING-R	association
MCG-L	paralimbic	ORBmid-L	paralimbic	PHG-L	paralimbic	SOG-L	association
MCG-R	paralimbic	ORBmid-R	paralimbic	PHG-R	paralimbic	SOG-R	association
PCG-L	paralimbic	IFGoperc-L	association	AMYG-L	limbic	MOG-L	association
PCG-R	paralimbic	IFGoperc-R	association	AMYG-R	limbic	MOG-R	association
PoCG-L	primary	IFGtriang-L	association	CAU-L	subcortical	IOG-L	association
PoCG-R	primary	IFGtriang-R	association	CAU-R	subcortical	IOG-R	association
SPG-L	association	ORBinf-L	paralimbic	PUT-L	subcortical	FFG-L	association
SPG-R	association	ORBinf-R	paralimbic	PUT-R	subcortical	FFG-R	association
IPL-L	association	SFGmed-L	association	PAL-L	subcortical	ANG-L	association
IPL-R	association	SFGmed-R	association	PAL-R	subcortical	ANG-R	association
SMG-L	association	ORBmed-L	paralimbic	THA-L	subcortical	MTG-L	association
SMG-R	association	ORBmed-R	paralimbic	THA-R	subcortical	MTG-R	association
PCUN-L	association	REC-L	paralimbic			ITG-L	association
PCUN-R	association	REC-R	paralimbic			ITG-R	association
PCL-L	association	ACG-L	paralimbic				
PCL-R	association	ACG-R	paralimbic				
HES-L	primary	TPOsup-L	paralimbic				
HES-R	primary	TPOsup-R	paralimbic				
STG-L	association	TPOmid-L	paralimbic				
STG-R	association	TPOmid-R	paralimbic				

*Tissue classification based on Mesulam (2000)

TABLE S5. Study inclusion and exclusion criteria	
**Inclusion criteria**	Healthy pregnancies with normal fetal echocardiogram and ultrasonogram
**Exclusion criteria**	Dysmorphic features by antenatal ultrasound
	Chromosomal abnormalities by amniocentesis
	Presentation after 28 weeks gestation
	Multiple gestations
	Evidence of congenital infections
